# Testosterone does not mediate the correlation between dietary inflammation and serum klotho levels among males: insights from NHANES database

**DOI:** 10.3389/fendo.2024.1370457

**Published:** 2024-04-02

**Authors:** Siyu Du, Jieyi Zhao, Xinyue Chou, Jingyu Peng, Qi Cao, Yimiao Zeng, Lu Ao, Xiaoyu Wang

**Affiliations:** ^1^ Department of Reproductive Medical Center, West China Second University Hospital, Sichuan University, Chengdu, Sichuan, China; ^2^ Key Laboratory of Birth Defects and Related Diseases of Women and Children (Sichuan University), Ministry of Education, Sichuan University, Chengdu, Sichuan, China; ^3^ West China School of Medicine, Sichuan University, Chengdu, Sichuan, China; ^4^ Department of Neurosurgery, West China Hospital, Sichuan University, Chengdu, Sichuan, China; ^5^ Innovation Institute, China Medical University, Shenyang, Liaoning, China; ^6^ West China School of Public Health and West China Fourth Hospital, Sichuan University, Chengdu, Sichuan, China

**Keywords:** klotho, testosterone, dietary inflammatory index, NHANES, male, mediating effect

## Abstract

**Introduction:**

Serum Klotho (S-Klotho) is a transmembrane protein holds pivotal roles in anti-aging. The Dietary Inflammation Index (DII), a meticulously dietary tool, quantifies the inflammatory potential of an individual's diet. The existing research strongly suggests that a low DII diet plays a significant role in delaying aging and reducing aging-related symptoms in males. Testosterone could potentially act as a mediating intermediary between DII and S-Klotho. However, this aspect remains unexplored. This study aims to investigate the potential causal link of testosterone between DII and S-Klotho in males.

**Methods:**

We utilized data from National Health and Nutrition Examination Survey (NHANES) which focused on male participants from 2013-2016. Mediation analyses were used to investigate the effects of testosterone (TT), free testosterone (FT), and free androgen index (FAI) on the DII-S-Klotho relationship, using three modes adjusting for covariates.

**Results:**

Mediation analysis unveiled a significant inverse correlation between DII and S-Klotho levels (model 1: c = -14.78, p = 0.046). The interaction between DII and S-Klotho was modulated by TT in model 1 (ab = -1.36; 95% CI: -5.59, -0.55; p = 0.008), but lost significance after adjustments (model 2: ab = -0.39; 95% CI: -4.15, 1.66; p = 0.378; model 3: ab = -0.59; 95% CI: -4.08, 2.15; p = 0.442). For FT, the mediating impact was not statistically significant (model 1: ab = 0.43; 95% CI: -0.51, 5.44; p = 0.188; model 2: ab = 0.72; 95% CI: -0.26, 5.91; p = 0.136; model 3: ab = 0.84; 95% CI: -0.02, 8.06; p = 0.056). Conversely, FAI consistently influenced the DII-S-Klotho relationship (model 1: ab = 2.39; 95% CI: 0.69, 9.42; p = 0.002), maintaining significance after adjustments (model 2: ab = 3.2; 95% CI: 0.98, 11.72; p = 0.004; model 3: ab = 3.15; 95% CI: 0.89, 14.51; p = 0.026).

**Discussion:**

This study observed no mediating influence of TT or FT on the correlation between DII and S-Klotho after covariate control. Remarkably, FAI continued to significantly mediate the DII-S-Klotho connection even following covariate adjustment, although its significance in males warrants careful consideration.

## Introduction

Serum Klotho (S-Klotho) is a transmembrane protein that plays pivotal roles in anti-aging and is considered a longevity-associated protein (1). The Klotho protein exists in two forms: membrane-bound and secretory. The detached membrane-bound variant is referred to as S-Klotho and can be detected in blood, urine, and cerebrospinal fluid (2, 3). Intriguingly, the dearth of Klotho gene expression in mice leads to a syndrome mirroring human aging, encompassing shortened lifespan, infertility, arteriosclerosis, skin atrophy, osteoporosis, and emphysema (4, 5). Conversely, overexpression of this protein appears to extend lifespan by 20-30% (6). In humans, plasma Klotho concentrations have demonstrated an inverse correlation with aging-related symptoms and overall mortality (7, 8).

The Dietary Inflammation Index (DII), a meticulously dietary tool, quantifies the inflammatory potential of an individual’s diet, has been shown to be highly correlated with six major inflammatory biomarkers across diverse populations (9–12). Emerging evidence suggests a crucial link between the DII and various health outcomes, particularly in relation to aging-related symptoms including mortality, cancer, cardiovascular diseases, musculoskeletal disorders, and mental well-being (1–4). The existing research strongly suggests that a low DII diet plays a significant role in delaying aging and reducing aging-related symptoms (13).

Population-based studies have also indicated a negative correlation between DII and S-Klotho (14). However, stratified analysis by gender revealed that this negative correlation is evident only in males (14). Considering the pivotal roles of sex hormones in metabolic and aging differences between males and females, and in light of existing research data, we hypothesize that sex hormones, particularly testosterone, may play an intermediary role in the negative correlation between DII and S-Klotho in the male population.

Mechanistically, testosterone activates klotho gene promoters through androgen receptor-mediated pathways, thereby enhancing klotho mRNA expression (15). Plausible mechanisms linking pro-inflammatory diets to diminished testosterone levels might involve pro-inflammatory markers such as increased IL-1, IL-6, IL-17, and TNF. Experimental evidence suggests that these markers impede testosterone secretion by influencing both central (hypothalamic-pituitary) and peripheral (testicular) components of the gonadal axis (16–18). In addition to the aforementioned mechanistic research, clinical studies from the United States (19–21) and Spain (22) have unveiled a negative correlation between DII and testosterone, while testosterone showed a positive association with S-Klotho in males. The amalgamation of these findings suggests that testosterone could conceivably serve as a mediating intermediary between DII and S-Klotho in adult males.

Notably, despite these intriguing possibilities, there is an evident dearth of research addressing this mediating role. Consequently, this study endeavors to explore the conceivable mediating function of testosterone within the complex interrelation between dietary inflammation and S-Klotho levels in adult males.

## Methods

### Study design and participants

In this cross-sectional study, we utilized data from NHANES (National Health and Nutrition Examination Survey), an ongoing nationwide survey conducted by the National Center for Health Statistics at the U.S. Centers for Disease Control and Prevention. NHANES aims to assess the nutritional and health status of the U.S. population and is conducted in two-year cycles with representative sample weights. The study protocol of NHANES has been approved by the institutional review board, and all participants provided written informed consent in accordance with the principles of the Declaration of Helsinki. The data used in our study are publicly available on the CDC (Centers for Disease Control and Prevention) website: https://www.cdc.gov/nchs/nhanes/.

**Figure 1:**
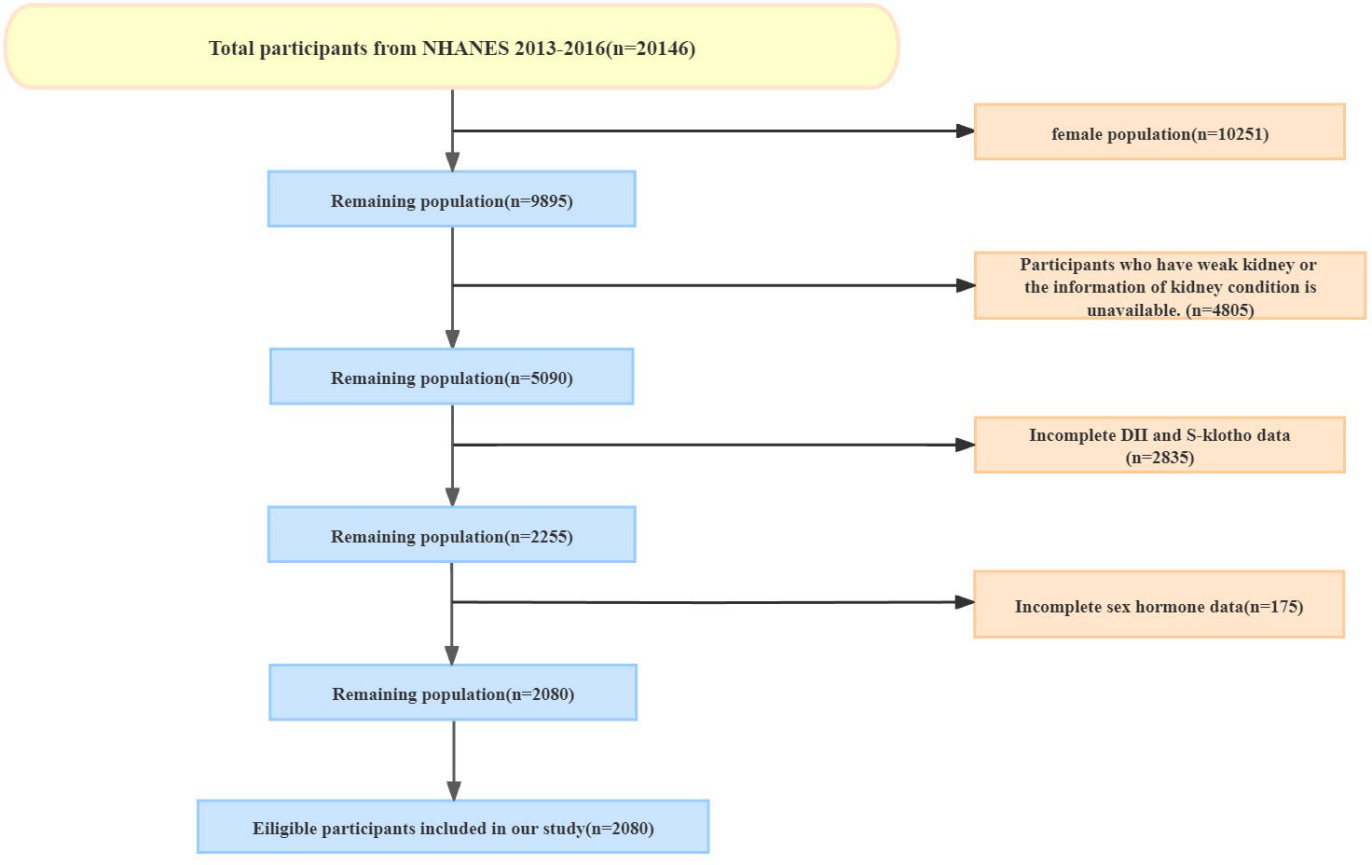
The participants enrollment. A total of 2080 people were included from NHANES (from 2013 to 2016).

We obtained the data from NHANES 2013-2016, which included comprehensive information on sex hormones, sex hormone-binding globulin (SHBG), dietary inflammatory index (DII) calculations, and serum Klotho (S-Klotho) levels. Our study focused on male participants who completed 24-hour dietary recall, underwent sex hormone and SHBG tests, as well as S-Klotho testing. If there was any absence of the information above, participants were excluded from our cohort. The detailed selection and exclusion procedure was as follows: (1) Exclude female participants. (2) Exclude male participants with kidney failure which is defined based on the question: ‘has he/she ever been told by a doctor or other health professional that had weak or failing kidneys’ in the questionnaire data or the information of kidney condition is unavailable. (3) Exclude participants with incomplete dietary data to calculate DII. (4) Exclude participants without S-Klotho serum levels. (5) Participants without testosterone test data or SHBG test data were excluded. This procedure is also presented in **Figure 1**.

### Dietary data and dietary inflammatory index

The dietary data used for calculating the DII were obtained from NHANES. The validity of these dietary data has been established by the Nutrition Methodology Working Group (23). We calculated the DII following the protocols described by Shivappa et al. (24). A lower DII score indicates a more anti-inflammatory diet, while a higher DII score suggests a more proinflammatory diet. Our study included 27 out of 45 food parameters available in the NHANES database, including protein, fat, alcohol, carbohydrates, fiber, cholesterol, omega-3 and omega-6 polyunsaturated fatty acids, saturated, monounsaturated, and polyunsaturated fatty acids, niacin, vitamin A, vitamin B1, vitamin B2, vitamin B6, vitamin B12, vitamin C, vitamin D, and vitamin E, iron, magnesium, zinc, selenium, folic acid, beta carotene, and caffeine. We treated the DII as a continuous variable and stratified it into four quarters for further statistical analysis.

### Testosterone, SHBG, FAI and FT

Testosterone (TT) levels were measured using the isotope dilution liquid chromatography tandem mass spectrometry (ID-LC-MS/MS) method, which is based on the reference method developed by the National Institute for Standards and Technology (NIST). The measurement of SHBG involved the reaction of SHBG with immuno-antibodies and chemoluminescence measurements of the reaction products, which occurred after two incubation periods and subjecting the samples to a magnetic field. The chemiluminescent reaction on the captured microparticles was measured using a photomultiplier tube. Detailed measurement protocols can be found at www.cdc.gov/nchs/nhanes/. Additionally, we calculated the free androgen index (FAI) by multiplying TT (ng/dL) by 100 and 0.288 and divided by SHBG (nmol/L), which provides an approximate estimation of the amount of circulating free testosterone (25). We also calculated the free testosterone (FT) based on the algorithm published by Vermeulen et al. (26).

### S-Klotho

Available pristine serum samples from 40-79 year old participants in NHANES were analyzed with the IBL ELISA method. The Northwest Lipid Metabolism and Diabetes Research Laboratories, Division of Metabolism, Endocrinology, and Nutrition, University of Washington, performed analyses on all but four fresh-frozen (pristine) samples received from the Centers for Disease Control and Prevention. All sample analyses were performed in duplicate according to the manufacturer’s protocol and all the results were checked to meet the laboratory’s standardized criteria for acceptability prior to being released for reporting.

### Covariates

Covariates in our study included age (in years), body mass index (BMI, in kg/m^2^), race/ethnicity (categorized as Mexican American, Other Hispanic, non-Hispanic White, non-Hispanic Black, or other race), ratio of family income to poverty (PIR), marital status (categorized as Married, Widowed, Divorced, Separated, Never married or Living with partner), smoking status, and alcohol intake. Smoking status was classified according to the NCHS classifications, where individuals who had smoked fewer than 100 cigarettes in their lifetime were considered never smokers, those who had smoked more than 100 cigarettes but were not currently smoking at the time of the survey were classified as former smokers, and those who had smoked more than 100 cigarettes in their lifetime and were currently smoking at the time of the survey were categorized as current smokers. Alcohol intake was categorized based on the question, “Have you had at least 12 alcohol drinks in the past year?” BMI was calculated using self-reported weight and height measurements and was categorized as underweight or healthy weight (<25 kg/m^2^), overweight (25-29.9 kg/m^2^), or obese (≥30 kg/m^2^) (19). PIR was categorized as low income (<1), middle income (1-4), or high income (≥4).

### Statistical analysis

We conducted the statistical analysis using R (version 3.5.3) and EmpowerStats (www.empowerstats.com; X&Y Solutions Inc.). Categorical variables were presented as percentages, while continuous variables were reported as means ± standard deviation. To analyze differences among the DII quarters, we employed weighted chi-square tests for categorical variables and weighted linear regression for continuous variables if the data fit the normal distribution. Otherwise, the weighted kruskal-wallis test will be used.

Mediation was estimated using the indirect effect, which represents the changes in the effect of the independent variable on the outcome that can be attributed to the proposed mediator (27). The “a” path represents effect of the independent variable on the mediator variable while the “b” path represents effect of the mediator variable on the dependent variable. Indirect effects (a × b paths) with confidence intervals that did not include zero were considered statistically significant which could occur regardless of the significance of the total effect (i.e. c path, effect of the independent variable on the dependent variable) and the direct effect (i.e. c’ path, effect on the dependent variable when both the independent and the mediator variables are included as independent variables) (27). To explore the effects of TT, SHBG and FAI on the relationship between DII and S-Klotho levels respectively, we performed weighted mediation analyses. Three models were used to assess the impact of covariates on this association: Model 1 included no covariate adjustments, Model 2 adjusted for age, race, and BMI group, and Model 3 adjusted for age, race, BMI group, Ratio of family income to poverty which is used as a categorical variable (low income: <1, middle income: 1-4, high income: ≥4), smoking status, alcohol drinking, and marital status. To quantify the magnitude of the total effect explained by mediation analysis, we calculated the percentage of mediation ([indirect effect/total effect] × 100) (28).

## Result

A total of 2080 males were included in the current analysis (**Figure 1**). The baseline characteristics of the participants are shown in **Table 1**. Participants adhering to the most anti-inflammatory diets (DII quarter 1) exhibited higher levels of S-Klotho (824.26 pg/mL vs. 798.52 pg/mL, p<0.001), free testosterone (7.08 ng/mL vs. 6.44 ng/mL, p=0.005), and free androgen index (35.42 vs. 31.15, p=0.001). and were more likely to have higher incomes (59.55% vs. 31.53%, p<0.001) and be non-smokers (56.33% vs. 44.42%, p<0.001) compared with participants with the most proinflammatory diets (DII quarter 4). The participants’ mean age was similar across DII quarters, as were testosterone, estradiol, SHBG, blood pressure, the proportions of BMI group, alcohol drinking and marital status.

**Table 1:** Characteristics of participants in NHANES 2013-2016 (weighted).

	DII-Q1	DII-Q2	DII-Q3	DII-Q4	*P-value*
N	520	520	520	520	
DII ^a^	-1.80 ± 0.85	-0.05 ± 0.35	1.25 ± 0.38	2.73 ± 0.57	<0.001
Klotho (pg/ml) ^a^	824.26 ± 300.48	809.81 ± 239.90	802.28 ± 261.55	798.52 ± 257.17	<0.001
Total testosterone (ng/dL) ^a^	421.21 ± 178.40	400.91 ± 160.25	417.72 ± 186.02	401.57 ± 172.98	0.121
Free testosterone (ng/dL) ^a^	7.08 ± 3.62	6.77 ± 2.46	6.86 ± 2.47	6.44 ± 2.68	0.005
Estradiol (pg/mL) ^a^	24.78 ± 10.81	24.82 ± 8.30	25.09 ± 9.30	25.40 ± 9.46	0.721
SHBG (nmol/L) ^a^	47.06 ± 22.63	45.93 ± 23.03	47.63 ± 25.36	49.27 ± 23.73	0.166
FAI ^a^	35.42 ± 24.46	33.78 ± 14.07	33.75 ± 13.47	31.15 ± 12.96	0.001
Age ^a^	56.25 ± 9.79	56.61 ± 10.53	56.81 ± 10.43	56.70 ± 10.65	0.82
Blood pressure (mmHg) ^a^					
SBP	125.38 ± 16.67	124.85 ± 14.77	126.72 ± 16.19	128.24 ± 17.73	0.006
DBP	73.46 ± 12.02	72.10 ± 11.18	73.29 ± 10.95	72.81 ± 12.89	0.234
BMI group (%)					0.063
Underweight or healthy weight	21.58	21.47	18.08	16.82	
Overweight	42.24	41.54	41.14	38.07	
Obese	36.18	36.98	40.78	45.11	
Race (%)					<0.001
Mexican American	6.41	9.00	7.13	6.33	
Other Hispanic	4.99	4.76	4.84	5.31	
Non-Hispanic White	75.46	71.11	73.83	69.93	
Non-Hispanic Black	5.69	5.33	8.45	13.19	
Other Race - Including Multi-Racial	7.45	9.80	5.74	5.24	
PIR (%)					<0.001
Low income	8.20	6.78	11.50	14.60	
Middle income	32.25	43.08	43.94	53.87	
High income	59.55	50.14	44.56	31.53	
Smoking (%)					<0.001
Never	56.33	47.42	42.99	44.42	
Former	33.14	39.76	32.37	34.10	
Current	10.52	12.82	24.64	21.48	
Alcohol drinking (%)					0.051
Yes	88.08	89.06	88.71	83.75	
No	11.92	10.94	11.29	16.25	
Marital status(%)					0.124
Married	66.67	71.04	68.37	61.07	
Widowed	2.13	2.08	4.25	2.84	
Divorced	9.22	11.04	12.10	15.34	
Separated	2.83	2.08	2.50	3.69	
Never married	9.91	9.25	7.41	7.95	
Living with partner	9.24	4.41	5.37	9.11	

a Mean ± SD.

Smoking status: individuals who had smoked fewer than 100 cigarettes in their lifetime were considered never smokers, those who had smoked more than 100 cigarettes but were not currently smoking at the time of the survey were classified as former smokers, and those who had smoked more than 100 cigarettes in their lifetime and were currently smoking at the time of the survey were categorized as current smokers. Alcohol intake was categorized based on the question, “Have you had at least 12 alcohol drinks in the past year?” BMI group: underweight or healthy weight (<25 kg/m^2^), overweight (25-29.9 kg/m^2^) or obese (≥30 kg/m^2^). PIR was categorized as low income (<1), middle income (1-4), or high income (≥4).

BMI, body mass index; DII, Dietary Inflammatory Index; PIR, Ratio of family income to poverty; NHANES, National Health and Nutrition Examination Survey; SBP, systolic blood pressure; DBP, diastolic blood pressure; FAI, free androgen index; SHBG, sex hormone-binding globulin.

**Figure 2:**
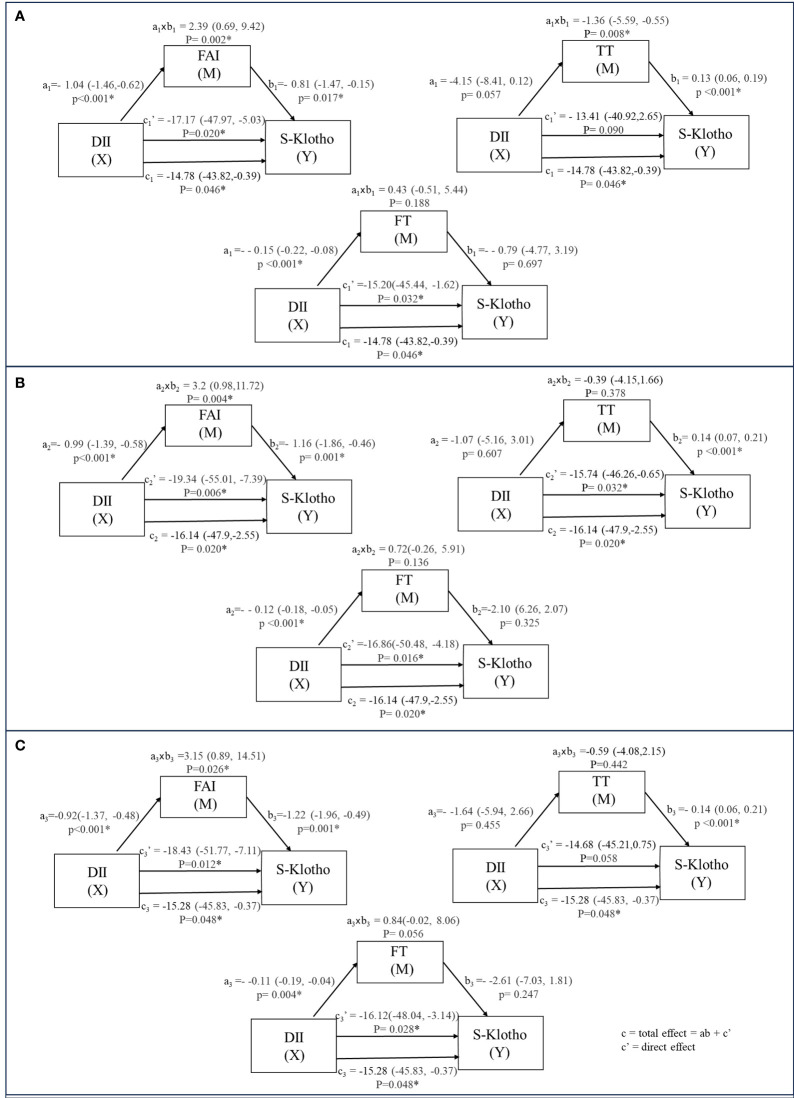
The relation between DII and S-Klotho through the indirect effect of TT, FT and FAI (weighted). a: Coefficients of the relationship between intermediary variable and independent variable. b: Coefficients of the relationship between implicit variable and intermediary variable. axb: Value of intermediary effect. **(A)** model 1 unadjusted. **(B)** model 2 adjusted for Age, Race, BMI group. **(C)** model 3 adjusted for Age; Race; BMI group; Ratio of family income to poverty; Smoking status; Alcohol drinking; Marital status.

The results indicated an inverse correlation between DII and S-2Klotho plasma levels (model 1: c = -14.78; 95% CI: -43.82, -0.39; p = 0.046; model 2: c = -16.14; 95% CI: -47.9, -2.55; p = 0.020; model 3: c = -15.28; 95% CI: -45.83, -0.37; p = 0.048) (**Figure 2**). Mediation analyses were carried out to test whether the association of DII with S-Klotho levels in males could be mediated by TT, FT or FAI respectively (**Tables 2**, **3**).

**Table 2:** Mediation analysis of the estimated effect of Dietary Inflammatory Index on the S-Klotho concentrations through Testosterone, FAI and FT among males in NHANES 2013-2016 (weighted).

	*β (95% CI)*	*P-value*	*β (95% CI)*	*P-value*	*β (95% CI)*	*P-value*
	TT		FT		FAI	
Model 1						
Total effect	-14.78 (-43.82,-0.39)	0.046*	-14.78 (-43.82, -0.39)	0.046*	-14.78 (-43.82, -0.39)	0.046*
Mediation effect	-1.36 (-5.59,-0.55)	0.008*	0.43(-0.51, 5.44)	0.188	2.39 (0.69, 9.42)	0.002*
Direct effect	-13.41 (-40.92,2.65)	0.090	-15.20(-45.44, -1.62)	0.032*	-17.17 (-47.97, -5.03)	0.020*
Propotion mediated	9.23%		-3.00%		-16.20%	
Model 2						
Total effect	-16.14 (-47.9,-2.55)	0.020*	-16.14 (-47.9, -2.55)	0.020*	-16.14 (-47.9, -2.55)	0.020*
Mediation effect	-0.39 (-4.15,1.66)	0.378	0.72(-0.26, 5.91)	0.136	3.2 (0.98,11.72)	0.004*
Direct effect	-15.74 (-46.26,-0.65)	0.032*	-16.86(-50.48, -4.18)	0.016*	-19.34 (-55.01, -7.39)	0.006*
Propotion mediated	2.44%		-4.00%		-19.85%	
Model 3						
Total effect	-15.28 (-45.83,-0.37)	0.048*	-15.28 (-45.83, -0.37)	0.048*	-15.28 (-45.83, -0.37)	0.048*
Mediation effect	-0.59 (-4.08,2.15)	0.442	0.84(-0.02, 8.06)	0.056	3.15 (0.89,14.51)	0.026*
Direct effect	-14.68 (-45.21,0.75)	0.058	-16.12(-48.04, -3.14)	0.028*	-18.43 (-51.77, -7.11)	0.012*
Propotion mediated	3.89%		-6.00%		-20.62%	

Model 1 unadjusted.

Model 2 adjusted for Age, Race, BMI group.

Model 3 adjusted for Age; Race; BMI group; PIR; Smoking status; Alcohol drinking; Marital status.

TT, total testosterone; FT, free testosterone; FAI, free androgen index; BMI, body mass index; PIR, Ratio of family income to poverty; NHANES, National Health and Nutrition Examination Survey.

BMI group: underweight or healthy weight (<25 kg/m^2^), overweight (25-29.9 kg/m^2^) or obese (≥30 kg/m^2^).

PIR: low income (<1), middle income (1-4), or high income (≥4).*: The statistical effect is significant.

**Table 3:** Mediation analysis of the estimated effect of Dietary Inflammatory Index on the serum S-Klotho concentrations through Testosterone, FAI and FT among males in NHANES 2013-2016 (weighted).

	*β (95% CI)*	*P-value*	*β (95% CI)*	*P-value*	*β (95% CI)*	*P-value*
	TT		FT		FAI	
Model 1						
a	-4.15 (-8.41, 0.12)	0.057	-0.15 (-0.22, -0.08)	<0.001*	-1.04 (-1.46,-0.62)	<0.001*
b	0.13 (0.06, 0.19)	<0.001*	-0.79 (-4.77, 3.19)	0.697	-0.81 (-1.47, -0.15)	0.017*
axb	-1.36 (-5.59, -0.55)	0.008	0.43 (-0.51, 5.44)	0.188	2.39 (0.69, 9.42)	0.002*
Model 2						
a	-1.07 (-5.16, 3.01)	0.607	-0.12 (-0.18, -0.05)	<0.001*	-0.99 (-1.39, -0.58)	0.001*
b	0.14 (0.07, 0.21)	<0.001*	-2.10 (6.26, 2.07)	0.325	-1.16 (-1.86, -0.46)	0.001*
axb	-0.39 (-4.15,1.66)	0.378	0.72 (-0.26, 5.91)	0.136	3.2 (0.98, 11.72)	0.004*
Model 3						
a	-1.64 (-5.94, 2.66)	0.455	-0.11 (-0.19, -0.04)	0.004*	-0.92 (-1.37, -0.48)	<0.001*
b	0.14 (0.06, 0.21)	<0.001*	-2.61 (-7.03, 1.81)	0.247	-1.22 (-1.96, -0.49)	0.001*
axb	-0.59 (-4.08,2.15)	0.442	0.84 (-0.02, 8.06)	0.056	3.15 (0.89, 14.51)	0.026*

a: Coefficients of the relationship between intermediary variable and independent variable.

b: Coefficients of the relationship between implicit variable and intermediary variable.

axb: Value of intermediary effect.

Model 1 unadjusted.

Model 2 adjusted for Age, Race, BMI group.

Model 3 adjusted for Age; Race; BMI group; PIR; Smoking status; Alcohol drinking; Marital status.

TT, total testosterone; FT, free testosterone; FAI, free androgen index; BMI, body mass index; PIR, Ratio of family income to poverty; NHANES, National Health and Nutrition Examination Survey.

BMI group: underweight or healthy weight (<25 kg/m^2^), overweight (25-29.9 kg/m^2^) or obese (≥30 kg/m^2^).

PIR: low income (<1), middle income (1-4), or high income (≥4).*: The statistical effect is significant.

Regarding TT, we found that the relation between DII and S-Klotho levels was significantly operated indirectly through TT (model 1: ab = -1.36; 95%Cl: -5.59, -0.55; p = 0.008), with a percentage of mediation of 9.23%. However, this mediating effect lost significance after adjustments (model 2: ab = -0.39; 95%Cl: -4.15,1.66; p = 0.378; model 3: ab = -0.59; 95%Cl: -4.08,2.15; p = 0.442). There was no significant association between TT and DII(model 1: a = -4.15; 95%Cl: -8.41, 0.12; p = 0.057; model 2: a = -1.07 95%Cl:-5.16, 3.01; p = 0.607; model 3: a = -1.64; 95%Cl: -5.94, 2.66; p = 0.455), but a positive direct association between TT and S-Klotho was observed (model 1: b = 0.13; 95%Cl: 0.06, 0.19; p <0.001; model 2: b = 0.14 95%Cl:0.07, 0.21; p <0.001; model 3: b = 0.14; 95%Cl: 0.06, 0.21; p<0.001).

For FT, the mediation analysis did not support a significant indirect influence of FT on the DII-S-Klotho relationship (model 1: ab = 0.43; 95%Cl: -0.51, 5.44; p = 0.188; model 2: ab = 0.72; 95%Cl: -0.26, 5.91; p = 0.136; model 3: ab = 0.84; 95%Cl: -0.02, 8.06; p = 0.056). There was a negative association between FT and DII(model 1: a = -0.15; 95%Cl: -0.22, -0.08; p <0.001; model 2: a = -0.12 95%Cl:-0.18, -0.05; p <0.001; model 3: a = -0.11; 95%Cl: -0.19, -0.04; p = 0.004), but no significant direct association was found between FT and S-Klotho levels (model 1: b =-0.79; 95%Cl: -4.77, 3.19; p = 0.697; model 2: b =-2.10 95%Cl:-6.26, 2.07; p =0.325; model 3: b = -2.61; 95%Cl: -7.03, 1.81; p= 0.247).

In the case of FAI, the mediation analysis established a significant indirect influence of FAI on the DII-S-Klotho relationship (model 1: ab = 2.39; 95%Cl: 0.69, 9.42; p = 0.002) and maintained stability after adjusted (model 2: ab = 3.2; 95%Cl: 0.98, 11.72; p = 0.004; model 3: ab = 3.15; 95%Cl: 0.89, 14.51; p = 0.026) with a percentage of mediation of -16.20%, -19.85% and -20.62% respectively. There was a negative association between FAI and DII(model 1: a = -1.04; 95%Cl: -1.46,-0.62; p <0.001; model 2: a = -0.99 95%Cl:-1.39, -0.58; p <0.001; model 3: a = -0.92; 95%Cl: -1.37, -0.48; p <0.001), as well as a negative direct association between FAI and S-Klotho levels (model 1: b =-0.81; 95%Cl: -1.47, -0.15; p = 0.017; model 2: b =-1.16; 95%Cl: -1.86, -0.46; p = 0.001; model 3: b = -1.22; 95%Cl: -1.96, -0.49; p= 0.001).

## Discussion

The study conducted mediation analyses to explore the potential impacts of testosterone (TT), free testosterone (FT), and free androgen index (FAI) on the relationship between the dietary inflammatory index (DII) and S-Klotho levels among males aged 40-79, using data from NHANES 2013-2014 and 2015-2016. Initially, a notable mediation effect was observed in the case of TT, indicating that the link between DII and S-Klotho levels was indirectly influenced by TT. This mediating influence accounted for a percentage of mediation. However, subsequent adjustments rendered this mediation effect statistically non-significant. While no direct significant correlation emerged between TT and DII, a positive direct association was detected between TT and S-Klotho levels. Conversely, the mediation analysis pertaining to FT failed to provide substantial support for an indirect impact of FT on the DII-S-Klotho relationship. Concurrently, a negative connection between FT and DII was established, though no significant direct association surfaced between FT and S-Klotho levels. On the other hand, the investigation into FAI yielded noteworthy outcomes, revealing a significant indirect influence of FAI on the DII-S-Klotho relationship through mediation. This mediating effect remained consistent even after adjustments were made and was quantified as a percentage of mediation. The alignment of our unadjusted findings with our hypothesis indicated that testosterone could potentially act as a causal mediator between DII and S-Klotho. Nevertheless, this effect weakened after accounting for covariates, possibly due to confounding factors such as Body Mass Index (BMI). Prior research, including systematic reviews and meta-analyses, has illuminated the strong connection between a pro-inflammatory diet and heightened annual weight gain, as well as a higher risk of developing overweight or obesity (29–31). Given the association between obesity and increased levels of various inflammatory markers, the presence of chronic low-grade inflammation in obesity can impact the production of testosterone and Sex Hormone-Binding Globulin (SHBG) through the hypothalamic-pituitary-gonadal axis (32–34). Studies on elderly males from the United States (35, 36) and Europe (37) have consistently demonstrated that obesity, as defined by parameters such as waist circumference, waist-to-hip ratio, or BMI, is correlated with declining levels of total and free testosterone.

The free hormone hypothesis emphasizes the biological activity of hormones based on free hormone concentrations in the plasma, rather than protein-bound hormones (38). Notably, observational studies within the EMAS cohort have underscored the importance of calculated Free Testosterone (FT) serum levels in understanding symptoms related to androgen deficiency in males (39). Contrary to expectations, our study showed that the mediating effect of FT on the DII-S-Klotho association did not reach statistical significance, but displayed a trend towards significance after controlling for confounding factors (p = 0.056). This intriguing finding suggests the potential for an independent mediating role of FT, warranting further investigation. The interplay between sex hormones, inflammatory markers, DII, and S-Klotho might involve complex interactions influenced by multifactorial conditions, necessitating comprehensive research for clarification.

Significantly, our study substantiated a robust mediating impact of the Free Androgen Index (FAI) on the DII-S-Klotho relationship, a result that persisted across model adjustments. FAI, albeit correlated with FT, remains distinct due to its dependence on measurements of TT and SHBG (40, 41). Despite its wide usage in clinical practice for assessing FT status in females, the applicability of FAI for estimating FT in males is limited due to assumptions inherent in its formula that are not applicable to males (42–44). As such, the statistical significance of FAI’s mediating effect should be interpreted with caution, as it might suggest an independent mediating role of FT in the DII-S-Klotho association.

The strengths of our study include the utilization of a nationally representative population and a relatively large sample size, enhancing the reliability and generalizability of our findings. Additionally, we employed liquid chromatography-tandem mass spectrometry (LC-MS/MS) for precise measurement of TT and the Vermeulen calculator for FT estimation, both of which improve accuracy and reliability. However, limitations must also be acknowledged. The cross-sectional nature of our study restricts causal inferences, necessitating prospective research. Furthermore, the reliance on 24-hour dietary recalls introduces recall bias, potentially affecting the DII and testosterone relationship. Additionally, measuring plasma testosterone at a single time point might not capture its dynamic fluctuations over time. The absence of crucial medical data, such as the usage of testosterone inhibitors, and a more precise assessment of potential confounding factors (e.g., alcohol intake assessment through the Alcohol Use Disorders Identification Test), could also introduce bias into the results.

## Conclusion

In conclusion, the present study unveiled intricate connections among dietary inflammation, sex hormones, and S-Klotho levels, highlighting the mediating effects mediated by testosterone. Although the mediating effect of TT no longer had statistical significance after accounting for covariates, a positive association with S-Klotho levels remained evident. FT did not exhibit a significant mediating effect. Notably, the considerable mediating effect of FAI on the relationship between the DII and S-Klotho persisted even after adjusting for covariates. However, its significance warrants careful consideration due to its limited effect in males. These findings underscore the imperative for future investigations aimed at unraveling the intricate mechanisms that underlie the dynamic interplay between inflammation, hormonal factors, and the intricate processes related to aging.

## Data availability statement

The datasets presented in this study can be found in online repositories. The names of the repository/repositories and accession number(s) can be found in the article/supplementary material.

## Ethics statement

The studies involving humans were approved by NHANES Institutional Review Board. The studies were conducted in accordance with the local legislation and institutional requirements. Written informed consent for participation was not required from the participants or the participants’ legal guardians/next of kin in accordance with the national legislation and institutional requirements. Written informed consent was obtained from the individual(s) for the publication of any potentially identifiable images or data included in this article.

## Author contributions

SD: Writing – original draft, Writing – review & editing. JZ: Writing – original draft, Writing – review & editing. XC: Writing – original draft, Writing – review & editing. JP: Data curation, Writing – original draft. QC: Writing – original draft, Writing – review & editing. YZ: Formal analysis, Writing – original draft, Writing – review & editing. AL: Conceptualization, Writing – original draft. XW: Funding acquisition, Writing – original draft.
